# Serum Copper, Zinc, and Iron Levels in Patients with Alzheimer's Disease: A Meta-Analysis of Case-Control Studies

**DOI:** 10.3389/fnagi.2017.00300

**Published:** 2017-09-15

**Authors:** Dan-Dan Li, Wei Zhang, Zhan-You Wang, Pu Zhao

**Affiliations:** ^1^College of Life and Health Sciences, Northeastern University Shenyang, China; ^2^Department of Hepatobiliary Surgery, General Hospital of Shenyang Military Area Command Shenyang, China

**Keywords:** copper, zinc, iron, Alzheimer's disease, meta-analysis

## Abstract

**Background:** Many publications have investigated the association between metal ions and the risk of Alzheimer's disease (AD), but the results were ambiguous.

**Aims:** The objective of this study was to assess the association between the serum levels of metals (copper/zinc/iron) and the risk of AD via meta-analysis of case-control studies.

**Methods:** We screened literatures published after 1978 in the Pubmed, Embase, Cochrane library, Web of Science and ClinicalTrials.gov. Electronic databases. By using Preferred Reporting Items for Systematic reviews and Meta-Analyses (PRISMA) guidelines, we performed a systematic review of the 407 publications, there are 44 of these publications met all inclusion criteria. The Review Manager 5.3 software was used to calculate available data from each study.

**Results:** Consistent with the conclusions of other meta-analysis, our results demonstrated serum copper levels were significantly higher [MD = 9.27, 95% CI (5.02–13.52); *p* < 0.0001], and the serum zinc levels were significantly lower in AD patients than in healthy controls [MD = −6.12, 95% CI (−9.55, −2.69); *p* = 0.0005]. Serum iron levels were significantly lower in AD patients than in healthy controls after excluded two studies [MD = −13.01, 95% CI (−20.75, −5.27); *p* = 0.001].

**Conclusion:** The results of our meta-analysis provided rigorous statistical support for the association of the serum levels of metals and the risk of AD, suggesting a positive relationship between the serum copper levels and AD risk, and a negative relationship between the serum zinc/iron levels and AD risk.

## Introduction

Alzheimer's disease (AD) is a progressive neurodegenerative disorder that leads to intellectual decline including memory loss, and language breakdown (International, 2009)^1^. Besides cognitive decline, AD patients have many other different manifestations, including mood disturbance and psychological symptoms (Paulsen et al., 2000; Tractenberg et al., 2002).

The deposition of β-amyloid peptide (Aβ) in the brain is one of the pathology hallmarks of AD (Citron, 2010; Buendia et al., 2016; Sepulcre et al., 2016; Kreutzer et al., 2017). Although high concentrations of endogenous copper (Cu) (Pal et al., 2015; Greenough et al., 2016; Xu et al., 2016), zinc (Zn) (Kulikova et al., 2015; Mezentsev et al., 2016), and iron (Fe) (Guo et al., 2015; Peters et al., 2015; Sands et al., 2016; James et al., 2017) have been found in these amyloidal plaques, the association of those metal ions with Aβ accumulation has not been well established. Recent studies (Exley, 2006; Alimonti et al., 2007; Azhdarzadeh et al., 2013; McCord and Aizenman, 2014; Yuan et al., 2014; Koç et al., 2015; Paglia et al., 2016) indicated that these bioactive metals are definitely important for the function of the brain, and are critical for Aβ aggregation and reactive oxygen species (ROS) production in the brains of AD patients and AD mouse model. For example, lacking of Zn can cause neuronal death and mild cognitive impairment (MCI) which occurs in elderly people (Sparks et al., 2006; Brewer, 2012). And for Cu, it can interact with Aβ, mediates the aggregation of Aβ under slightly acidic conditions, and facilitates the generation of oxidative stress (Cheignon et al., 2016). For Fe, it is another essential ion that participates the cellular processes of neurons, and its importance was recently being realized by scientific communities, as it can catalyze fenton-based reactions to generate ROS (Gonzalez-Dominguez et al., 2014). Studies showed that Fe can accumulate in Aβ plagues (Shore et al., 1984; Jeandel et al., 1989; Molaschi et al., 1996; Gonzalez et al., 1999) and neurons with neurofibrillary tangles (Gonzalez-Dominguez et al., 2014), bind with the iron-responsive element RNA stem loop in the 5′-UTR of amyloid-β protein precursor (AβPP) mRNA, and then regulate the translation of AβPP (Molina et al., 1998; Squitti et al., 2013b) and AD progression.

On the basis of these evidences, the potential role of Cu/Zn/Fe dysfunction in the pathogenesis of AD has been the object of much investigation over the past decades. Therefore, the aim of our study was to provide more useful information about the relationship between the serum levels of Cu/Zn/Fe and AD susceptibility by carried out the present meta-analysis of case-control studies published in the past few years on the role of Cu, Zn, and Fe in AD.

## Materials and methods

### Search strategy

We searched for case-control studies articles published from 1978 to May 2016 via systematically screening in the PUBMED, EMBASE, Cochrane library, Web of science and clinical trials.gov electronic databases according to PRISMA guidelines (Moher et al., 2010) by using the following search terms In the keywords “Alzheimer's disease”(or “AD”), “copper” (or “Cu” or “Cu^2+^”), “zinc” (or “Zn” or “Zn^2+^”), “iron” (or “Fe” or “Fe^2+^”), “metals.” Additional studies were obtained from the reference lists of identified studies.

### Selection criteria

According to PRISMA guideline (Moher et al., 2010), we choose the following inclusion criteria for the meta-analyses: (1) full-text publications written in English; (2) case-control studies about the association of Cu, Zn, and Fe with the AD; (3) studies providing the serum level of Cu, Zn, and Fe to calculate the mean difference (MD) or standardized mean difference (SMD) and 95% confidence intervals (CIs).

Studies were excluded for the following reasons: (1) non-case-control studies trials, such as case reports, reviews, and meta-analysis; (2) family-based studies; (3) without original data; (4) non-human; and (5) were published in a language other than English.

### Study selection and data extraction

Studies were identified by two independent authors using the aforementioned search strategy. When there was uncertainty regarding eligibility, the other two authors were consulted. The following information was collected from each study: name of the first author; year of publication; country; criteria for AD diagnosis, sample sizes of patients and controls, mean age, and percentage of females in groups. We assessed how metal levels were measured with the following data: type of sample; assay method; laboratory or kit used; collection, process, and storage of sample; blinding of laboratory personnel; and the use of quality control sample. Data in other forms [i.e., odds ratio and mean ± 95% confidence interval (CI)] were converted to mean ± SD according to the Cochrane Handbook (Higgins and Green, 2009). If data were not reported numerically, we extracted them by manual measurements from published figures.

### Statistical analysis

The meta-analyses were done using the Review Manager 5.3 software. Total MD with 95% CIs were determined to evaluate the strength of the association between Cu/Zn/Fe and AD risk. Heterogeneity was assessed with the Cochran Q-statistic and the I^2^ test (Coory, 2010). Studies with an I^2^ value < 25% were considered to have low heterogeneity, and those with an I^2^ >75% were considered to have a high degree of heterogeneity. If there is *P*_Q_ > 0.05 among the involved studies in the meta-analysis, the fixed-effect model is used; otherwise, the random-effect model is adopted. If there is significant heterogeneity, we omitted each individual study in turn from the total, and reanalyzed the remainder. All statistical analyses were conducted with Review Manager 5.3 software and the two-sided *P* < 0.05 in the *Z*-test was deemed to be statistically significant.

## Results

### Study identification and selection

**Figure 1:**
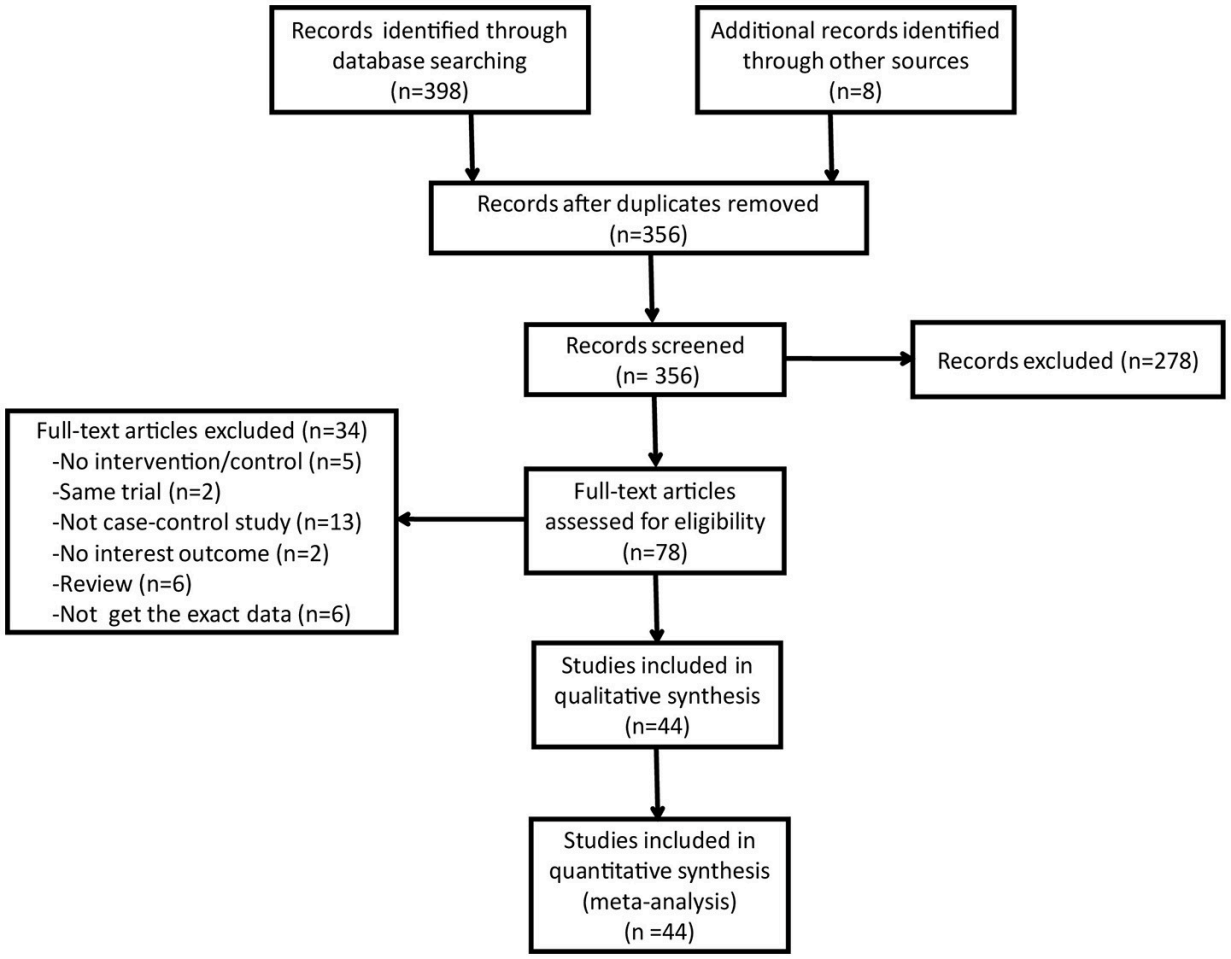
Study selection for systematic review.

The procedures for the study selection are displayed in Figure 1. The initial search yielded 406 relevant publications form the PUBMED, EMBASE, Cochrane library, Web of science and clinical trials.gov. Duplicate references were automatically removed. The title and abstract of the remaining 356 publications were evaluated according to predefined exclusion and inclusion criteria. Then, 278 publications were excluded, 34 were subsequently excluded and 44 (Shore et al., 1984; Jeandel et al., 1989; Kapaki et al., 1989; Molaschi et al., 1996; Thome et al., 1996; Molina et al., 1998; Gonzalez et al., 1999; Maes et al., 1999; Ozcankaya and Delibas, 2002; Squitti et al., 2002a,b, 2005, 2006, 2007, 2010, 2011, 2013a,b; Smorgon et al., 2004; Bocca et al., 2005; Sedighi et al., 2006; Alimonti et al., 2007; Sevym et al., 2007; Agarwal et al., 2008; Dong et al., 2008; Zappasodi et al., 2008; Baum et al., 2010; Brewer et al., 2010a,b; Giambattistelli et al., 2012; Mueller et al., 2012; Azhdarzadeh et al., 2013; Huang et al., 2013; Lopez et al., 2013; Rembach et al., 2013, 2014; Crespo et al., 2014; Gonzalez-Dominguez et al., 2014; Park et al., 2014; Singh et al., 2014; Koç et al., 2015; Wang et al., 2015; Paglia et al., 2016; Siotto et al., 2016) were included in the systematic review and meta-analysis.

### Studies on Cu in serum

**Figure 2:**
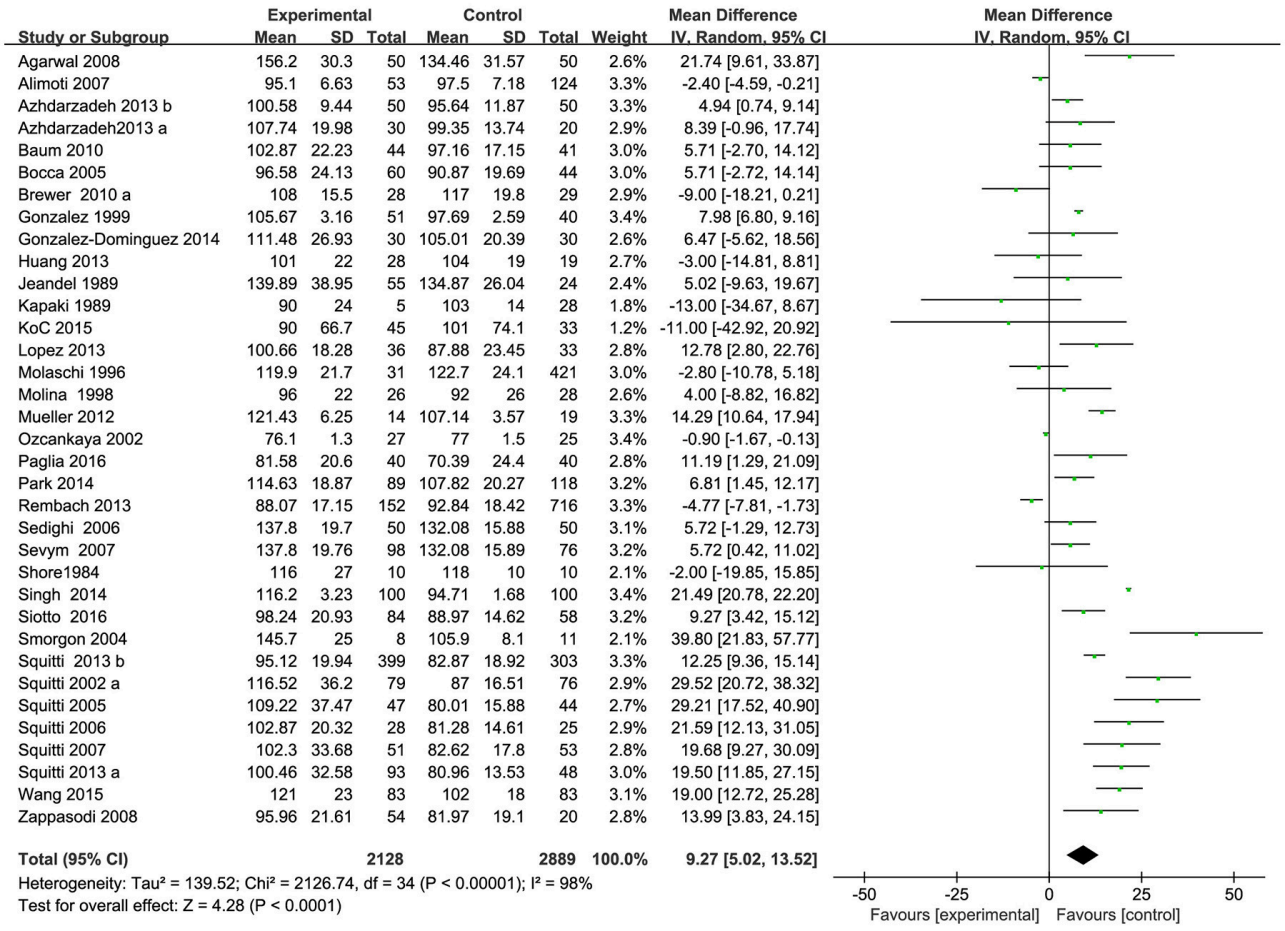
Forest plots for serum Cu levels in AD and healthy controls in included studies.

Characteristics of the 35 included studies are summarized in Table 1. As show in Figure 2, 35 form 34 articles with a total of 2,128 AD patients and 2,889 healthy controls were included in serum Cu meta-analysis. To note, the paper by Azhdarzadeh et al. (2013) consisted of two study groups and each group was detected separately. The patient sample sizes of the included studies are ranged from 5 (Kapaki et al., 1989) to 399 (Squitti et al., 2013b) and control sample sizes from 10 (Shore et al., 1984) to 716 (Rembach et al., 2013). The mean age of the patient groups was >54. The percentage of female AD patients ranged from 20% (Kapaki et al., 1989) to 100% (Molaschi et al., 1996).

**Table 1:** Studies included in the meta-analysis of serum copper levels.

**Study**	**Country**	**Alzheimer's disease**					**Health controls**					**Publication quality assessment**	***P*-value**
		***N***	**Female (%)**	**Mean age (y)**	**MMSE**	**Converted serum level (ug/dl)**	***N***	**Female (%)**	**Mean age (y)**	**MMSE**	**Converted serum level (ug/dl)**		
Siotto et al., 2016	Italy	84	0.69	77 ± 8.75	< 25	98.24 ± 20.93	58	0.47	64.5 ± 18	< 25	88.97 ± 14.62	^******^	*p* = 0.51
Paglia et al., 2016	Italy	34	0.74	72.44 ± 7.48	12.31 ± 8.15	81.58 ± 20.6	40	0.625	65.53 ± 6.37	29.57 ± 0.75	70.39 ± 24.4	^*****^	*p* = 0.039
Koç et al., 2015	Turkey	45	0.49	77.66 ± 9.29	–	90 ± 66.7	33	0.51	73.18 ± 10.61	–	101 ± 74.1	^*****^	*p* = 0.1
Wang et al., 2015	China	83	0.64	73.99 ± 7.13	–	121 ± 23	83	0.63	72.24 ± 7.48	–	102 ± 18	^***^	*p* < 0.05
Gonzalez-Dominguez et al., 2014	Spain	30	0.60	80.9 ± 4.5	–	111.48 ± 26.93	30	0.57	74.0 ± 5.7	–	105.01 ± 20.39	^*****^	NS
Singh et al., 2014	India	100	0.39	62.74 ± 7.20	–	116.20 ± 3.23	100	0.39	59.71 ± 8.11	–	94.71 ± 1.68	^******^	*p* < 0.001
Park et al., 2014	Korea	89	0.54	77.83 ± 6.65	–	114.63 ± 18.87	118	0.58	69.93 ± 5.89	–	107.82 ± 20.27	^******^	*P* = 0.049
Azhdarzadeh et al., 2013	Hong Kong	30	–	–	–	107.74 ± 19.98	20	–	–	–	99.35 ± 13.74	^*****^	NS
Azhdarzadeh et al., 2013	Iran	50	–	–	–	100.58 ± 9.44	50	–	–	–	95.64 ± 11.87	^*****^	*p* < 0.005
Squitti et al., 2013a	Italy	93	0.77	75.14 ± 8.69	18.98 ± 5.18	100.46 ± 32.58	48	0.48	70.29 ± 8.98	–	80.96 ± 13.53	^******^	*p* = 0.002
Squitti et al., 2013b	Italy	399	0.32	74.9 ± 8.1	19.5 ± 4.5	95.12 ± 19.94	303	0.32	66.5 ± 10.5	28.6 ± 1.3	82.87 ± 18.92	^******^	*p* < 0.001
Lopez et al., 2013	Spain	36	0.55	77.75 ± 5.31	20.7 ± 4.4	100.66 ± 18.28	33	0.64	74.00 ± 5.03	28.9 ± 1.3	87.88 ± 23.45	^***^	*p* = 0.038
Rembach et al., 2013	Australia	152	0.59	77 ± 7.9	18.9 ± 5.3	88.07 ± 17.15	716	0.58	69 ± 6.8	28.9 ± 1.2	92.84 ± 18.42	^*****^	NS
Huang et al., 2013	Taiwan	28	0.86	83.00 ± 6.8	15.64 ± 4.22	101 ± 22	19	0.74	79.89 ± 7.0	23.06 ± 5.51	104 ± 19	^*******^	*p* = 0.560
Mueller et al., 2012	USA	14	0.64	80.6 ± 2.7	–	121.43 ± 6.25	19	0.47	75.6 ± 4.0	–	107.14 ± 3.57	^****^	NS
Brewer et al., 2010a	USA	28	0.46	76.2	24.0 ± 3.91	108 ± 15.5	29	0.69	68.6	29.8 ± 0.69	117 ± 19.8	^******^	*p* = 0.05
Baum et al., 2010	Hong Kong	44	0.66	74.3 ± 8.7	< 30	102.87 ± 22.23	41	0.49	79.1 ± 6	< 30	97.16 ± 17.15	^******^	NS
Zappasodi et al., 2008	Italy	54	0.81	73.7 ± 8.7	19.5 ± 3.8	95.96 ± 21.61	20	0.65	71.55 ± 9.2	28 ± 1.7	81.97 ± 19.1	^****^	*p* = 0.021
Agarwal et al., 2008	India	50	0.38	59.96 ± 11.57	14.07 ± 7.59	156.2 ± 30.30	50	0.34	55.32 ± 10.88	–	134.46 ± 31.57	^******^	*p* = 0.002
Sevym et al., 2007	Turkey	98	0.66	72.1 ± 6.7	–	137.80 ± 19.76	76	0.59	70.3 ± 5.7	–	132.08 ± 15.89	^****^	*p* = 0.001
Alimonti et al., 2007	Italy	53	0.68	74.5 ± 6.5	17 ± 6.5	95.1 ± 6.63	124	0.35	44.8 ± 12.7	–	97.5 ± 7.18	^****^	–
Squitti et al., 2007	Italy	51	0.78	73 ± 8	19.2 ± 4.2	102.3 ± 33.68	53	0.66	70 ± 10	28.5 ± 1.2	82.62 ± 17.8	^******^	*p* < 0. 001
Sedighi et al., 2006	Iran	50	0.48	76.4	14.3 ± 4.6	137.80 ± 19.7	50	0.50	67.8	25.8 ± 1.5	132.08 ± 15.88	^******^	NS
Squitti et al., 2006	Italy	28	0.71	71.4 ± 8.6	15.5 ± 6.2	102.87 ± 20.32	25	0.68	70 ± 9.6	28.4 ± 1.2	81.28 ± 14.61	^****^	*p* < 0.001
Bocca et al., 2005	Italy	60	0.67	74.6 ± 6.39	2–28	96.58 ± 24.13	44	0.75	>45	–	90.87 ± 19.69	^*******^	NS
Squitti et al., 2005	Italy	47	0.74	75.6 ± 7.7	18.6 ± 4.7	109.22 ± 37.47	44	0.45	71.1 ± 11	28.1 ± 1.3	80.01 ± 15.88	^*****^	*p* < 0.001
Smorgon et al., 2004	Italy	8	–	79 ± 5	–	145.7 ± 25	11	–	78 ± 9	–	105.9 ± 8.1	^****^	*p* < 0.001
Ozcankaya and Delibas, 2002	Turkey	27	0.29	72.3 ± 6.5	16.8 ± 1.3	76.1 ± 1.3	25	0.36	64.4 ± 7.2	28.2 ± 2.4	77.0 ± 1.5	^*****^	NS
Squitti et al., 2002a	Italy	79	0.68	74.5 ± 7.4	17.3 ± 4.9	116.52 ± 36.20	76	0.57	70.1 ± 10.8	27.7 ± 2.2	87.0 ± 16.51	^******^	*p* < 0.001
Gonzalez et al., 1999	Spain	51	0.71	74.5 ± 2.3	–	105.67 ± 3.16	40	0.45	70.3 ± 4.0	–	97.69 ± 2.59	^*****^	*p* = 0.048
Molina et al., 1998	Spain	26	0.46	73.1 ± 68.2	13.2 ± 5.7	96 ± 22	28	0.43	70.8 ± 67.3	–	92 ± 26	^*****^	NS
Molaschi et al., 1996	Italy	31	1	77.2 ± 2.4	–	119.9 ± 21.7	421	1	77.6 ± 2.3	–	122.7 ± 24.1	^***^	NS
Jeandel et al., 1989	France	55	0.73	81.7 ± 5.7	< 25	139.89 ± 38.95	24	0.40	–	–	134.87 ± 26.04	^***^	NS
Kapaki et al., 1989	Iran	5	0.2	54	–	90 ± 24	28	0.36	46	–	103 ± 14	^****^	NS
Shore et al., 1984	USA	10	0.3	63.7 ± 8.4	–	116 ± 27	10	0.7	61.9 ± 8.0	–	118 ± 10	^***^	NS

* *Newcastle-Ottawa Scale is used to assess the quality of publications. The maximum number of stars is seven. MMSE, Mini Mental State Examination; p-value, the significance reported by the authors in their studies relatively to the comparison for copper, zinc, and iron between AD patients and healthy controls; NS, not significant*.

**Figure 3:**
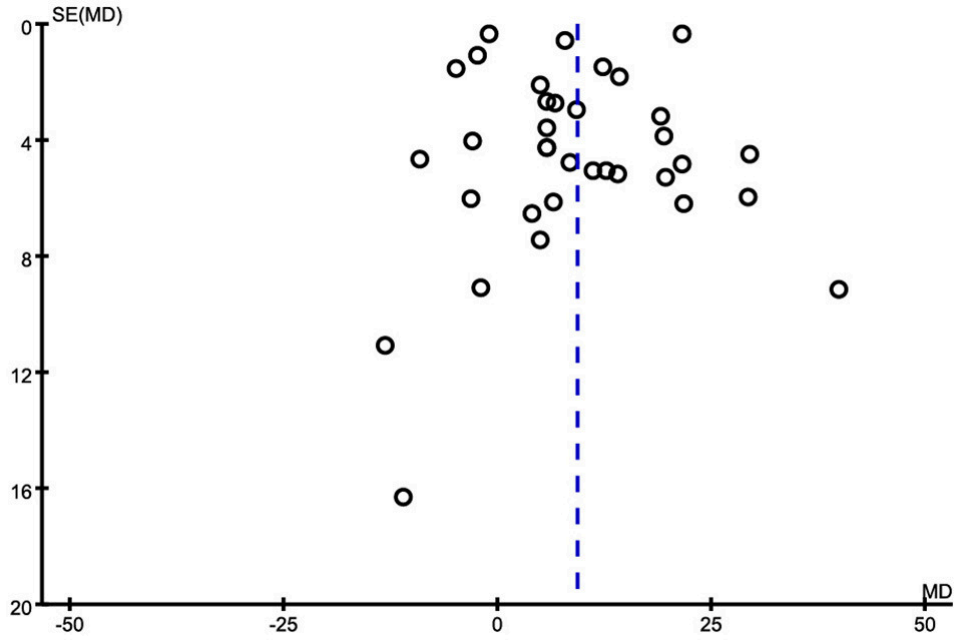
Funnel plot for publication bias in the selection of studies on serum Cu.

Of the 35 included studies, results are discordant, as shown in Table 1, 26 studies reported an increase of serum Cu levels in AD patients. The other 9 studies reported a decrease of serum Cu levels in AD patients, but two of the studies reported a tiny increase. Combined analysis of the relationship between the serum Cu level and AD was shown in a forest plot (Figure 2). The meta-analysis demonstrated Cu levels were significantly higher in AD patients than controls [MD = 9.27, 95% CI (5.02–13.52); *p* < 0.0001]. As a high heterogeneity between the included studies was observed (I^2^ = 98%), we ran a sensitivity analysis and found that the I^2^ = 89% after excluding the study of Singh et al. (2014) and Ozcankaya and Delibas (2002). After the exclusion, the result of this meta-analysis showed that serum Cu levels were significant higher in AD patients [MD = 9.13; 95% CI (6.17, 12.09); *p* < 0.00001]. Publication bias was assessed graphically using a funnel plot (Figure 3). Moreover, we ran a subgroup analysis for the country (*p* = 0.13), the mean age of the subjects (*p* = 0.66), and the percentage of women (*p* = 0.18) as a possible confounder, which revealed no variation of serum Cu level strictly associated with them.

### Studies on Zn in serum

**Figure 4:**
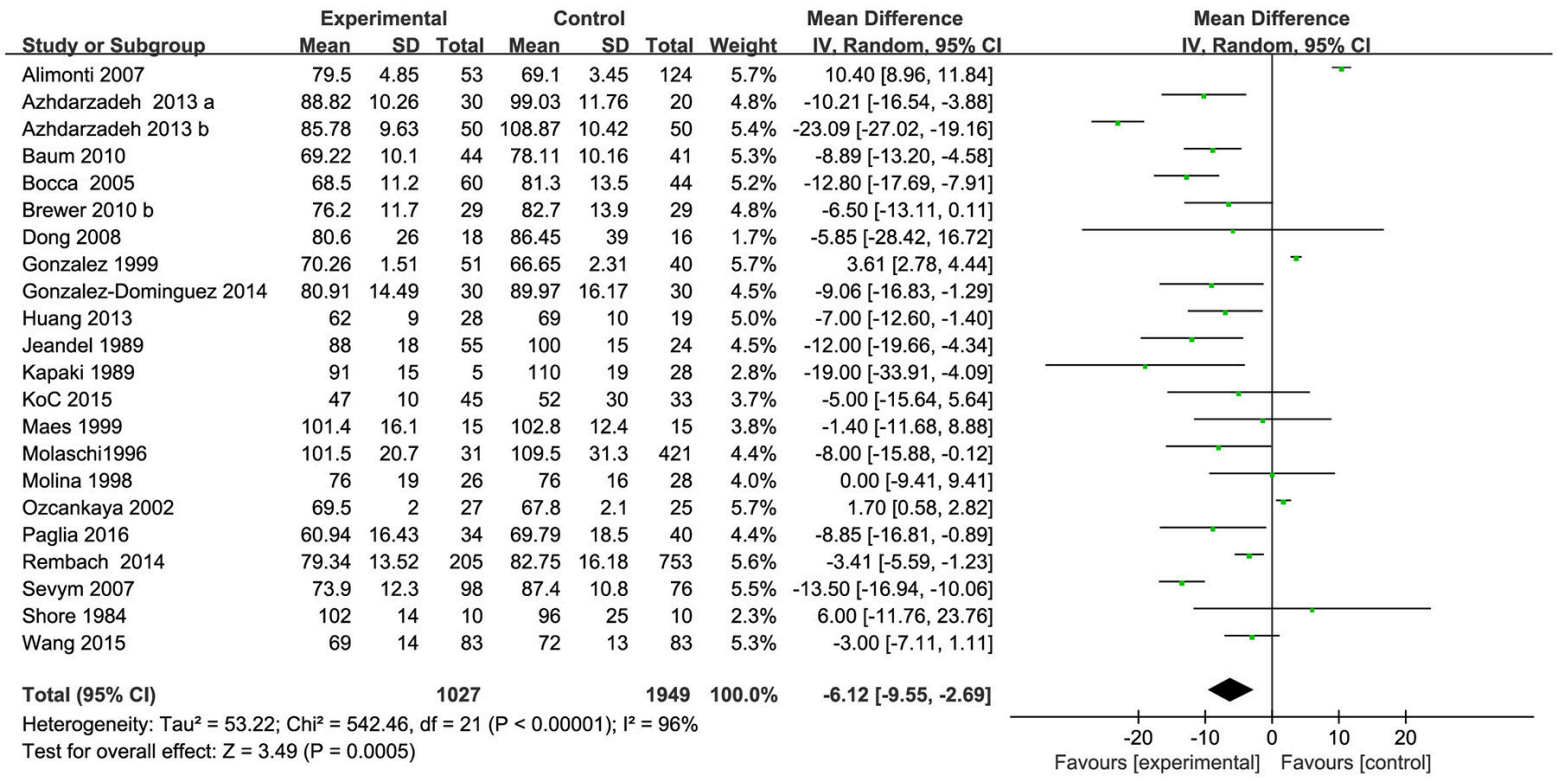
Forest plots for serum Zn levels in AD and healthy controls in included studies.

Characteristics of the 22 included studies are summarized in Table 2. As shown in Figure 4, 22 form 21 articles with a total of 1,027 AD patients and 1,949 healthy controls were included in serum Zn meta-analysis. The patient sample sizes ranged from 5 (Kapaki et al., 1989) to 205 (Rembach et al., 2014) and control sample sizes from 10 (Shore et al., 1984) to 753 (Rembach et al., 2014). The mean age of the patient groups was >54. The percentage of female AD patients ranged from 20% (Kapaki et al., 1989) to 100% (Molaschi et al., 1996).

**Table 2:** Studies included in the meta-analysis of serum zinc levels.

**Study**	**Country**	**Alzheimer's disease**					**Health controls**					**Publication quality assessment**	***P*-value**
		**N**	**Female (%)**	**Mean age (y)**	**MMSE**	**Converted serum level (ug/dl)**	**N**	**Female (%)**	**mean age (y)**	**MMSE**	**Converted serum level (ug/dl)**		
Paglia et al., 2016	Italy	34	0.74	72.44 ± 7.48	12.31 ± 8.15	60.94 ± 16.43	40	0.625	65.53 ± 6.37	29.57 ± 0.75	69.79 ± 18.5	^*****^	*p* = 0.020
Koç et al., 2015	Turkey	45	0.49	77.66 ± 9.29	–	47 ± 10	33	0.51	73.18 ± 10.61	–	52 ± 30	^*****^	*p* = 0.4
Wang et al., 2015	China	83	0.64	73.99 ± 7.13	–	69 ± 14	83	0.63	72.24 ± 7.48	–	72 ± 13	^***^	NS
Gonzalez-Dominguez et al., 2014	Spain	30	0.60	80.9 ± 4.5	–	80.91 ± 14.49	30	0.57	74.0 ± 5.7	–	89.97 ± 16.17	^*****^	NS
Rembach et al., 2014	Australia	205	0.62	78.8 ± 8.6	18.9 ± 5.3	79.34 ± 13.52	753	0.58	70.6 ± 7	28.9 ± 1.2	82.75 ± 16.18	^*****^	*p* < 0.01
Azhdarzadeh et al., 2013	Hong Kong	30	–	–	–	88.82 ± 10.26	20	–	–	–	99.03 ± 11.76	^*****^	*p* < 0.05
Azhdarzadeh et al., 2013	Iran	50	–	–	–	85.78 ± 9.63	50	–	–	–	108.87 ± 10.42	^*****^	*p* = 0.001
Huang et al., 2013	Taiwan	28	0.86	83.00 ± 6.8	15.64 ± 4.22	62 ± 9	19	0.74	79.89 ± 7.0	23.06 ± 5.51	69 ± 10	^*******^	*p* = 0.043
Baum et al., 2010	Hong Kong	44	0.66	74.3 ± 8.7	< 30	69.22 ± 10.1	41	0.49	79.1 ± 6.0	< 30	78.11 ± 10.16	^******^	*p* < 0.001
Brewer et al., 2010b	USA	29	0.43	73.5 ± 7.8	24.4 ± 4	76.2 ± 11.7	29	0.69	60.8 ± 13.9	–	82.7 ± 13.9	^******^	*p* = 0.027
Dong et al., 2008	USA	18	0.50	80.3 ± 1.7	18.0 ± 2.1	80.6 ± 26	16	0.44	77.9 ± 1.7	28.8 ± 0.4	86.45 ± 39	^****^	*p* < 0.05
Sevym et al., 2007	Turkey	98	0.66	72.1 ± 6.7	–	73.90 ± 12.30	76	0.59	70.3 ± 5.7	–	87.40 ± 10.80	^***^	*p* = 0.001
Alimonti et al., 2007	Italy	53	0.68	74.5 ± 6.5	17 ± 6.5	79.5 ± 4.85	124	0.35	44.8 ± 13	–	69.1 ± 3.45	^****^	–
Bocca et al., 2005	Italy	60	0.67	74.6 ± 6.39	2–28	68.5 ± 11.2	44	0.33	>45	–	81.3 ± 13.5	^*******^	*p* < 0.05
Ozcankaya and Delibas, 2002	Turkey	27	0.30	72.3 ± 6.5	16.8 ± 13	69.5 ± 2.0	25	0.36	64.4 ± 7.2	28.2 ± 2.4	67.8 ± 2.1	^*****^	NS
Gonzalez et al., 1999	Spain	51	0.71	74.5 ± 2.3	–	70.26 ± 1.51	40	0.45	70.3 ± 4.0	–	66.65 ± 2.31	^*****^	NS
Maes et al., 1999	Belgium	15	0.80	78.4 ± 10.3	< 16	101.4 ± 16.1	15	0.47	75.6 ± 9.1	–	102.8 ± 12.4	^****^	NS
Molina et al., 1998	Spain	26	0.46	73.1 ± 68.2	13.2 ± 6	76 ± 19	28	0.43	70.8 ± 67.3	–	76 ± 16	^*****^	NS
Molaschi et al., 1996	Italy	31	1.00	77.2 ± 2.4	–	101.5 ± 20.7	421	1.00	77.6 ± 2.3	–	109.5 ± 31.3	^***^	NS
Kapaki et al., 1989	Iran	5	0.2	54.0 ± 2.0	–	91 ± 15	28	0.36	44.5 ± 14.3	–	110 ± 19	^****^	–
Jeandel et al., 1989	France	55	0.73	81.7 ± 5.7	< 25	88 ± 18	24	0.79	76.4 ± 6.1	–	100 ± 15	^***^	*p* < 0.01
Shore et al., 1984	USA	10	0.3	63.7 ± 8.4	–	102 ± 14	10	0.7	61.9 ± 8.0	–	96 ± 25	^***^	NS

* *Newcastle-Ottawa Scale is used to assess the quality of publications. The maximum number of stars is seven. MMSE, Mini Mental State Examination; p-value, the significance reported by the authors in their studies relatively to the comparison for copper, zinc, and iron between AD patients and healthy controls; NS, not significant*.

**Figure 5:**
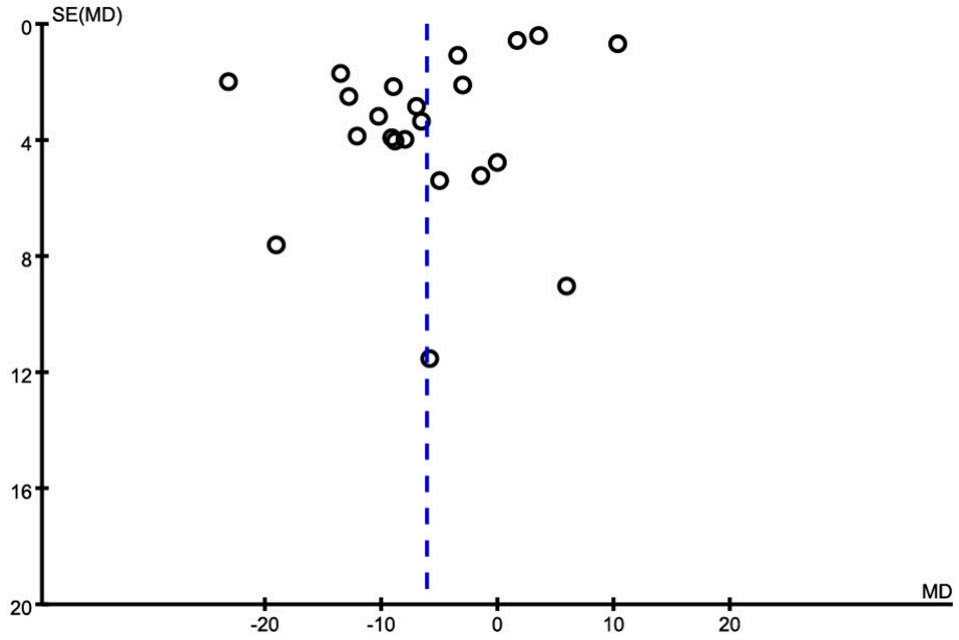
Funnel plot for publication bias in the selection of studies on the serum Zn.

Of the 22 included studies, results are discordant. As shown in Table 2, 3 studies reported an increase of serum Zn in AD patients; 18 studies reported a decrease, but none of them showed a significant difference of serum Zn between AD patients and controls; one of the studies reported that no significantly change of serum Zn was observed between AD patients and controls. Combined analysis of the relationship between the serum zinc level and AD was shown in a forest plot (Figure 2). The meta-analysis demonstrated Zn levels were significantly lower in AD patients than controls [MD = −6.12, 95% CI (−9.55, −2.69); *p* = 0.0005]. As a high heterogeneity between the included studies was observed (I^2^ = 96%), we ran a sensitivity analysis and found that the I^2^ = 92% after excluding the study of Alimonti et al. (2007) and Gonzalez et al. (1999). After the exclusion, significant differences of the serum Zn were observed between AD patients and normal controls [MD = −7.80, 95% CI (−11.61, −3.99); *p* < 0.0001]. Publication bias was assessed graphically using a funnel plot (Figure 5). Moreover, we also ran a subgroup analysis for the percentage of women (*p* = 0.11), the mean age (*p* = 0.55), and country (*p* = 0.05, I^2^ = 61.11%) as a possible confounder, which revealed no variation of the serum Zn strictly associated with them.

### Studies on Fe in serum

**Figure 6:**
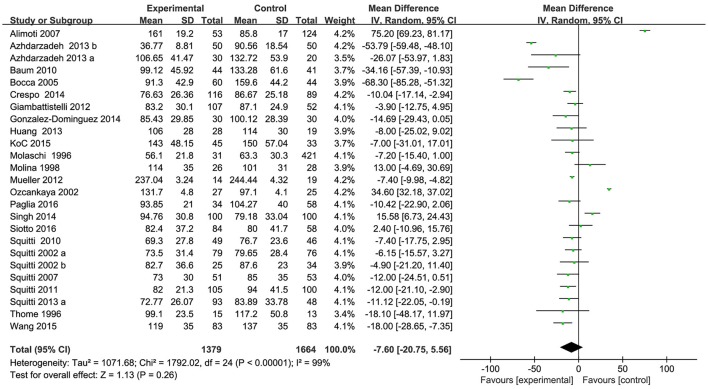
Forest plots for serum Fe levels in AD and healthy controls in included studies.

Characteristics of the 25 included studies are summarized in Table 3. As shown in Figure 6, 25 form 24 articles with a total of 1,379 AD patients and 1,664 healthy controls were included in serum Fe meta-analysis. The patient sample sizes are ranged from 14 (Mueller et al., 2012) to 116 (Crespo et al., 2014) and control sample size from 13 (Thome et al., 1996) to 421 (Molaschi et al., 1996). The mean age of the patient groups was >62.74. The percentage of female AD subjects ranged from 32% (Ozcankaya and Delibas, 2002) to 100% (Molaschi et al., 1996).

**Table 3:** Studies included in the meta-analysis of serum iron levels.

**Study**	**Country**	**Alzheimer's disease**					**Health controls**					**Publication quality assessment**	***P*-value**
		**N**	**Female (%)**	**Mean age (y)**	**MMSE**	**Converted serum level (ug/dl)**	**N**	**Female (%)**	**Mean age (y)**	**MMSE**	**Converted serum level (ug/dl)**		
Siotto et al., 2016	Italy	84	0.69	77 ± 8.75	< 25	82.4 ± 37.2	58	0.47	64.5 ± 18	< 25	80 ± 41.7	^******^	*p* = 0.06
Paglia et al., 2016	Italy	34	0.74	72.44 ± 7.48	12.31 ± 8.15	93.85 ± 21	40	0.625	65.53 ± 6.37	29.57 ± 0.75	104.5 ± 27.1	^*****^	*p* = 0.039
Koç et al., 2015	Turkey	45	0.49	77.66 ± 9.29	–	143 ± 48.15	33	0.51	73.18 ± 10.61	–	150 ± 57.04	^*****^	*p* = 0.2
Wang et al., 2015	China	83	0.64	73.99 ± 7.13	–	119 ± 35	83	0.63	72.24 ± 7.48	–	137 ± 35	^***^	*p* < 0.05
Crespo et al., 2014	Spain	116	0.79	76.6 ± 6.9	13.0 ± 6.1	76.63 ± 26.36	89	0.57	68.2 ± 7.7	28.8 ± 1.9	86.67 ± 25.18	^******^	*p* < 0.05
Gonzalez-Dominguez et al., 2014	Spain	30	0.60	80.9 ± 4.5	–	85.43 ± 29.85	30	0.57	74.0 ± 5.7	–	100.12 ± 28.39	^*****^	NS
Singh et al., 2014	India	100	0.39	62.74 ± 7.20	–	94.76 ± 30.80	100	0.47	59.71 ± 8.11	–	79.184 ± 33.04	^******^	*p* < 0.05
Azhdarzadeh et al., 2013	Hong Kong	30	–	–	–	106.65 ± 41.47	20	–	–	–	132.72 ± 53.9	^*****^	*p* < 0.05
Azhdarzadeh et al., 2013	Iran	50	–	–	–	36.77 ± 8.81	50	–	–	–	90.56 ± 18.54	^*****^	NS
Huang et al., 2013	Taiwan	28	0.86	83.00 ± 6.8	15.64 ± 4.22	106 ± 28	19	0.74	79.89 ± 7.0	23.06 ± 5.51	114 ± 30	^*******^	*p* = 0.428
Squitti et al., 2013a	Italy	93	0.77	75.14 ± 8.69	18.98 ± 5.18	72.77 ± 26.07	48	0.48	70.29 ± 8.98	28.6 ± 1.3	83.89 ± 33.78	^******^	NS
Mueller et al., 2012	USA	14	0.64	80.6 ± 2.7	–	237.04 ± 3.24	19	0.47	75.6 ± 4.0	–	244.44 ± 4.32	^****^	NS
Giambattistelli et al., 2012	Italy	107	–	75 ± 7.8	28.10 ± 1.2	83.2 ± 30.1	52	–	65 ± 10.3	19.9 ± 4.6	87.1 ± 24.9	^*****^	NS
Squitti et al., 2011	Italy	105	0.78	74 ± 8.0	19.6 ± 4.6	82 ± 21.3	100	0.57	69 ± 9.7	28.2 ± 1.2	94 ± 41.5	^******^	NS
Baum et al., 2010	Hong Kong	44	0.66	74.3 ± 8.7	< 30	99.12 ± 45.92	41	0.49	79.1 ± 6.0	< 30	133.28 ± 61.6	^******^	*p* < 0.001
Squitti et al., 2010	Italy	49	0.78	75.6 ± 7.7	19 ± 3.9	69.3 ± 27.8	46	0.46	71.2 ± 10.8	28.2 ± 1.2	76.7 ± 23.6	^******^	*p* = 0.209
Alimonti et al., 2007	Italy	53	0.68	74.5 ± 6.5	17 ± 6.5	161 ± 19.2	124	0.35	44.8 ± 12.7	–	85.8 ± 17.0	^****^	–
Squitti et al., 2007	Italy	51	0.78	73 ± 8	19.2 ± 4.2	73 ± 30	53	0.68	70 ± 10	28.5 ± 1.2	85 ± 35	^******^	*p* = 0.106
Bocca et al., 2005	Italy	60	0.67	74.6 ± 6.39	2–28	91.3 ± 42.9	44	0.33	>45	–	159.6 ± 44.2	^*******^	*p* < 0.05
Squitti et al., 2002b	Italy	25	0.76	78 ± 25	18.2 ± 5.2	82.7 ± 36.6	34	0.35	70 ± 11	29.1 ± 1	87.6 ± 23.0	^*******^	NS
Squitti et al., 2002a	Italy	79	0.68	74.5 ± 7.4	17.3 ± 4.9	73.5 ± 31.4	76	0.57	70.1 ± 10.8	27.7 ± 2.2	79.65 ± 28.4	^******^	NS
Ozcankaya and Delibas, 2002	Turkey	27	0.32	72.3 ± 6.5	16.8 ± 1.3	131.7 ± 4.8	25	0.57	64.4 ± 7.2	28.2 ± 2.4	97.1 ± 4.1	^*****^	*p* < 0.05
Molina et al., 1998	Spain	26	0.46	73.1 ± 68.2	13.2 ± 5.7	114 ± 35	28	0.43	70.8 ± 67.3	–	101 ± 31	^*****^	NS
Thome et al., 1996	Germany	15	–	69.7 ± 7.8	–	99.1 ± 23.5	13	–	69.7 ± 7.8	–	117.2 ± 50.8	^***^	NS
Molaschi et al., 1996	Italy	31	1	77.2 ± 2.4	18–25	56.1 ± 21.8	421	1	77.6 ± 2.3	–	63.3 ± 30.3	^***^	NS

* *Newcastle-Ottawa Scale is used to assess the quality of publications. The maximum number of stars is seven. MMSE, Mini Mental State Examination; p-value, the significance reported by the authors in their studies relatively to the comparison for copper, zinc, and iron between AD patients and healthy controls; NS, not significant*.

**Figure 7:**
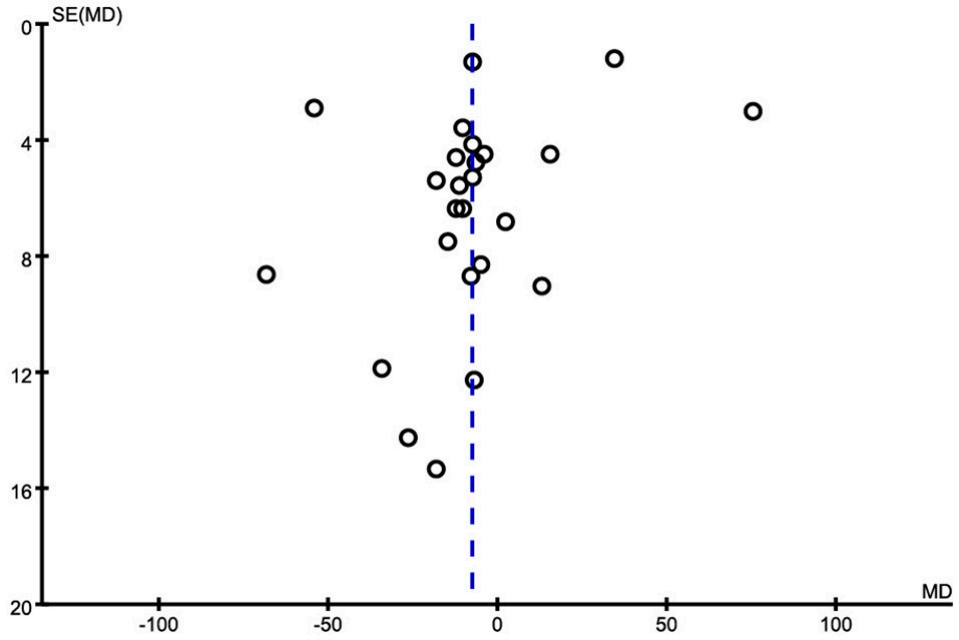
Funnel plot for publication bias in the selection of studies on the serum Fe.

Of the 25 included studies, results are discordant, as shown in Table 3, 5 studies reported an increase of serum Fe levels in AD patients, but one of the studies reported a tiny increase. The other 20 studies reported a decrease of serum Fe levels in AD patients. Combined analysis of the relationship between the serum Fe level and AD was shown in a forest plot (Figure 6). The results indicated that on significant difference of Fe levels were observed between AD patients and controls [MD = −7.60, 95% CI (−20.75, 5.56); *p* = 0.26]. As a high heterogeneity between the included studies was observed (I^2^ = 99%), we ran a sensitivity analysis and found that the I^2^ = 93% after excluding the study of Alimonti et al. (2007) and Ozcankaya and Delibas (2002). After the exclusion, significant differences of the serum Fe were observed between AD patients and normal controls [MD = −13.01, 95% CI (−20.75, −5.27); *p* = 0.001]. Publication bias was assessed graphically using a funnel plot (Figure 7). Moreover, we also ran a subgroup analysis for the percentage of women (*p* < 0.00001, I^2^ = 97.0%), the mean age (*p* = 0.31), and country (*p* = 0.68) as a possible confounder, which revealed no variation of the serum Fe strictly associated with them. From the above, we concluded that the variation of the serum Fe concentration found in some of the studies might be associated with the percentage of women of the subjects.

## Discussion

AD is the most common form of irreversible dementia, and is clinically characterized by the progressive memory problems. Abnormal homeostasis of trace metals is also observed in AD patients, especially the metabolism of Cu, Zn, and Fe. The purpose of this meta-analysis was to evaluate the association between serum Cu/Zn/Fe and AD risk. To do so, we performed a meta-analysis of selected case-control studies papers from 1975 to 2016 that enriched the literatures on these topics. We ran a meta-analysis for the serum Cu/Zn/Fe in AD patients and healthy controls in case-control studies.

### Serum Cu levels are positively and serum Zn levels are negatively associated with AD

Senile plaques are the typical pathology change of AD brains, which are composed of variety of components, including extracellular deposits of Aβ, lipids, heavy metal, and so on. Based on this evidence, Aβ plaques are sought to be a therapeutic targets of AD. In the past decades, several different classes of drugs were generated to prohibit Aβ plaques formation. But finally, many of them failed to improve the cognition of AD patients in the phase III clinical trials, suggesting that inhibition of senile plaques formation by using γ-secretase inhibitors or Aβ antibodies are not effective ways to dealing with AD. Another approach to reduce the toxicity of Aβ aggregation is to modulate the homeostasis of heavy metals, especially Cu, Zn, and Fe. Potential association between these metal ions and AD were showed. Dysfunction of metal ions could be an indicator for AD diagnosis and a good target for AD therapy.

*In vivo*, Cu was absorbed in the small intestine by amino acid transporters, and then was transported into the blood in two forms, ceruloplasmin (Cp) and non-Cp Cu. Non-Cp Cu is also called “free copper” which is more freely available for cellular uses. The study form Dr. Rosanna Squitti et al. has proved that “free Cu” level is elevated in AD patients when compared with age-matched controls (Squitti et al., 2005). Their recent meta-analysis study revealed that the increase in total Cu observed in AD patients can be attributed to the increase in “free Cu” in serum (Squitti et al., 2014), suggesting that the total Cu level in serum can be used to evaluate the bioavailable Cu in serum.

The conclusion of our meta-analysis revealed a significant increase of serum Cu and a decrease of serum Zn in AD patients than in healthy controls. In this study, we also ran a subgroup analysis for the possible confounders, including mean age of the subjects, percentage of women and the country. Differences of the serum Cu and Zn are unlikely to be affected by these factors, which suggesting that the overall results of our meta-analysis on Cu/Zn level were statistically robust. Focusing on the relationship between Cu and Zn, we found that these metal ions are competed with each other. They shared binding partners, such as melallothionein (MT), copper/zinc-superoxide dismutase 1 (SOD1), and Aβ. Zn-binding MT and the other Zn-binding proteins are function as a buffer system to buffer serum Cu, and then maintain the Cu homeostasis. Except for forming Zn binding proteins, Zn also mediated cell signaling pathways via modulating the activity of transcription factors, including Sp1, MTF-1, EGR1, GR, RAR, Ikaros, and Churchill. *In vitro*, MTs can bind more than seven equivalents of metal ions (Meloni et al., 2009; Sutherland et al., 2012). They adjust the balance of heavy metal ions, especially Cu and Zn. Generally speaking, when Cu^+^ levels is increased in cells, they competed the metal binding sites in MT and other metal binding proteins with Zn^2+^, thus increase free Zn^2+^ in cells. Free Zn^2+^ then bind with Zn-sensitive transcription factors (MTF-1), to activate the expression of MTs. Then the elevated MTs bind with Cu^+^ and take Cu^+^ out into the stool as intestinal cells are shed.

When Cu level is rising for a short term, the metal buffer system composed of MTs and metal-binding proteins can buffer the instantaneous increase of Cu^+^ effectively. But when Cu level is rising for a long term, cells will change their status to fit the Cu disorder. In this situation, metal binding protein, such as MTs (Hidalgo et al., 2006) and Cp (Park et al., 2014) are up-regulated to minimize the Cu toxicity. If the Cu level rise is beyond the body short-term buffer capacity (competing metal binding site in metalloproteins with Zn) and long-term buffer capacity (increasing metalloprotein expression), the Cu level is irreversible increasing in general circulation. These Cu could loosely bind to low-molecular-weight proteins or peptides, and cross the blood brain barrier to reach the brain. Then the overloaded Cu will enhance toxicity of Aβ via inducing ROS production in Aβ aggregation (Mayes et al., 2014). To validate the effects of Cp Cu and non-Cp Cu on AD progression, Dr. Rosanna Squitti and their collegues studied the correlationship of serum Cu in AD and Aβ concentration in cerebrospinal fluid (CSF). They found that “free Cu” in serum is negatively associated with Aβ in the CSF sustained the direct interaction between Cu^2+^ and Aβ (Squitti et al., 2006). Although, our meta-analysis results consistent with many of others' that serum Cu level is positively (Bucossi et al., 2011; Squitti et al., 2014; Kisler et al., 2017), and serum Zn level is negatively (Ventriglia et al., 2015; Wang et al., 2015) associated with AD risk, the causal relationship between Cu/Zn level and AD is still in debate. Fortunately, the epidemiologic data from using copper plumbing and AD, and the success application of Cu chelators and Zn reagents in mouse model of AD provide strong evidences that AD is a heavy metal overloading disease.

### Serum Fe was significantly lower in AD patients

Fe is another essential metal that plays a key role in AD pathophysiology. Similar as Cu, Fe has a high binding affinity to Aβ peptides, and it can provoke oxidative stress through the Fenton's reaction (Sayre et al., 2001). The postmortem analysis indicated that Fe co-localized with Aβ in senile plaques in AD brains (Jiang et al., 2009; Moreira et al., 2010). Moreover, the Fe-responsive 5′-UTR region in AβPP promoter is the potential target for Fe ions. The study from Crespo et al. indicated that transferrin and ferritin are significant decreased in the serum of AD patients than in controls. These two proteins are the key proteins that control the transportation and the storage of Fe in the body. It is also reported that the gene variation of transferrin and ferritin are associated with AD (Giambattistelli et al., 2012). According to these evidences, it is reasonable to speculated that Fe homeostasis may be associated with AD progression. The results of our meta-analysis revealed that no significant changes of serum Fe concentration were observed between AD patients and healthy controls, pointing out a high heterogeneity. But after the sensitivity analysis, we found that the studies of Alimonti et al. (2007) and Ozcankaya and Delibas (2002) have a big impact on the overall result. After excluding these two studies, the heterogeneity was reduced to 93.0% and serum Fe was significantly lower in AD patients (MD = −15.144; *p* = 0.002) which is consistent with previous meta-analysis resulted from Tao et al. (2014). We excluded the studies by Alimonti et al. (2007) because the age of some controls was < 45 years. The studies of Ozcankaya et al. showed that low levels of melatonin are associated with the development of AD, especially when accompanied with an increased Fe levels, so that might be the source of the heterogeneity of this study. Although the present meta-analysis showed that the serum Fe level was significantly lower in AD patients after excluding two studies with high heterogeneity, more studies are needed reveal the association of serum Fe and AD.

### Limitations

The present study still had some potential limitations that warrant mention. First, significant heterogeneity existed in the meta-analysis of the serum Cu/Zn/Fe in AD patients and healthy controls. The heterogeneity of the involved case-control studies are partially associated with the confounders, therefore, heterogeneity was still a problem that may affect the precision of the overall results in this meta-analysis. Second, the case-control studies means that the findings are might be affected by varying levels of bias owing to the quality evaluation of literature. Although the involved studies were reasonably homogeneous in most areas, there were some between-study variations that may have effects on the outcomes and thus the results of our meta-analysis. Third, we must acknowledge that the present case-control studies meta-analyses included limited number of studies; hence, the results must be clearly interpreted with some degree of caution. Fourth, only a few studies involving North America, Oceania were included in this meta-analysis. More studies are needed from other countries to evaluate the association between the serum metal levels and AD.

## Conclusion

In conclusion, from our current study, we provide statistical support that serum Cu was significantly increased and serum Zn/Fe was significantly decreased in AD patients. For serum Cu, the present case-control studies meta-analysis along with other four meta-analyses reached the same conclusion (Citron, 2010; Schrag et al., 2013; Squitti et al., 2014; Wang et al., 2015). For serum Zn, the results of our meta-analysis are consistent with other two meta-analyses (Ventriglia et al., 2015; Wang et al., 2015). For serum Fe, the results of the present meta-analysis are consistent with other two meta-analyses(Tao et al., 2014; Wang et al., 2015), indicating that serum Fe was significantly lower in AD patients than in healthy controls after excluding the studies of Alimonti et al. (2007) and Ozcankaya and Delibas (2002), but notably, this conclusion was not robust and needs further studies.

## Author contributions

PZ and ZW designed the study. DL and WZ performed the experiments and data analysis. PZ and WZ wrote the paper.

### Conflict of interest statement

The authors declare that the research was conducted in the absence of any commercial or financial relationships that could be construed as a potential conflict of interest. The reviewer BMM and handling Editor declared their shared affiliation.

## Footnotes

^1^International, A. S. D. (2009). World Alzheimer Report 2009.
